# Development of rapid multiplex human herpesvirus detection systems based on recombinase polymerase amplification and a lateral flow assay

**DOI:** 10.3389/fcell.2026.1751135

**Published:** 2026-05-26

**Authors:** Yidan Sun, Shuyu Ling, Dani Tang, Haoyi Zu, Meimei Yang, Chao Shen

**Affiliations:** 1 China Center for Type Culture Collection, Wuhan University, Wuhan, China; 2 Hubei Key Laboratory of Cell Homeostasis, College of Life Sciences, Wuhan University, Wuhan, China

**Keywords:** cell quality control, HHV, LFA, PCR, RPA

## Abstract

Human herpesviruses (HHVs) are highly prevalent globally (e.g., Epstein-Barr virus with over 95% prevalence) and can persist latently for life, causing severe conditions like blindness, encephalitis, and even lethal infections in neonates. HHV reactivation is of particular clinical concern in transplantation medicine, where immunosuppression can trigger viral reactivation, leading to graft rejection, secondary infections, and increased mortality. Notably, latently infected cells show no pathological changes, their contamination in cell cultures not only undermines the accuracy of scientific data and reliability of research results but also risks public health through the transmission of biological products. This study constructed three detection systems by combining multiplex Polymerase Chain Reaction (PCR) with Recombinase Polymerase Amplification (RPA) and Lateral Flow Assay (LFA): the Octaplex PCR system, with a sensitivity of 1 × 10^4^ copies per 50 μL system, suitable for high-throughput contamination screening of cell samples (1 out of 30 tested samples was EBV positive); the Quadruplex RPA system, which improved sensitivity to 1 × 10^3^ copies per 50 μL system (10 times higher than the PCR system), and shortened reaction time to 15–20 min, applicable for rapid preliminary screening; And the Duplex RPA-LFA detection system, maintaining 1 × 10^3^ copies per 50 μL system sensitivity, enabling visual result detection within 20 min without complex instruments for on-site rapid identification. These systems provide reliable tools for cell quality control, technical support for early diagnosis of HHVs-associated diseases and prevention strategies, as well as theoretical references for other virus detection systems, boasting broad application prospects.

## 1 Introduction

Cell culture is pivotal to biology, medicine, and drug development, yet contamination, by bacteria, fungi, *mycoplasma*, and viruses (Weiskirchen et al., 2023), poses a critical barrier. Among these, viral contamination is the most intractable: it is hard to detect/remove and causes long-term harm. This harm manifests most directly in research settings, where viral contamination can severely skew experimental outcomes. For instance, Influenza A virus (IAV) infecting A549 cells disrupts the JAK-STAT pathway via abnormal STAT1 phosphorylation (Pothlichet et al., 2013). Beyond compromising scientific integrity, improper handling of contaminated biological materials can trigger public health crises, as seen in the 1980s, when virus-contaminated plasma caused HIV/HCV infections and thousands of deaths in hemophiliacs (Weinberg et al., 2002). It arises from two main sources: 1) Cells isolated from virus-carrying tissues (undetectable with early technologies, amplifying risks); 2) Improper operations (e.g., inadequate utensil disinfection, non-sterile practices, or cross-contamination via shared reagents) (Merten, 2002).

Human Herpesviruses (HHVs) represent a particularly challenging group of viral contaminants in cell culture. These medium-sized, enveloped double-stranded DNA viruses have 8 human-infecting types, with a core trait: lifelong latency in host cells post-primary infection, reactivatable under stress (e.g., culture environment changes). Although HHVs are widespread in the general population, their latent and reactivatable nature poses specific challenges in research settings. Latency facilitates their silent introduction into cell banks via tissue-derived primary cells; reactivation disrupts cell metabolism, induces cytopathic effects (CPE) (Arvin et al., 2007), like Alphaherpesvirinae (HSV-1/2) cause rapid CPE in monolayers, or drives malignancy, like Gammaherpesvirinae (EBV) transforming lymphocytes (Choi and Lee, 2024). Both severely compromise experimental reliability and bioproduct (e.g., vaccines) safety.

Beyond the risk of single-virus contamination, HHVs often co-infect clinical tissues. More critically, co-infection often leads to synergistic interactions that exacerbate pathological damage. Among them, subtypes such as CMV and EBV often act synergistically (Frenzel et al., 2014), where CMV mainly damages epithelial cells and EBV targets lymphocytes, together causing complex and unpredictable interference. Studies have shown that co-reactivation of HHV-6 and CMV in ICU patients leads to poorer clinical outcomes than reactivation of either virus alone (Lopez Roa et al., 2015). Moreover, in liver transplantation, reactivation of HHV-6 and HHV-7 may indirectly worsen prognosis through their interaction with CMV (Razonable and Paya, 2002). Patients co-infected with HHV-5/6 show more frequent allograft rejection, secondary infections, and a higher risk of CMV disease (Sampaio et al., 2012). If the cell line used for research has potential multiple HHV infections, it may exert unpredictable and complex interference on the research results regarding immune responses, signaling pathways, etc., based on this cell model. Such unpredictable interference underscore that detecting a single virus is insufficient, as it fails to capture the complex interactions that can significantly worsen the impact on cell cultures, mirroring the worsened outcomes seen in clinical settings.

Consequently, the combination of their latent nature and co-infection potential makes multiplex detection not just beneficial but essential for comprehensive cell bank screening. This necessity is further amplified by the biological diversity among HHVs, which are classified into Alpha (1–3), Beta (5–7), and Gamma (4,8) subfamilies (Jia et al., 2025), each differs in latency sites (e.g., Alpha in ganglia, Beta in monocytes), replication cycles, and target cells (e.g., EBV in lymphocytes, HCMV in epithelial cells. A detection method capable of accounting for this variability is crucial for accurate risk assessment.

Current HHV detection methods have limitations: Virus isolation (HSV-1/2 gold standard) is time-consuming (Céspedes-Tenorio and Arias-Arias, 2023); serological tests (ELISA, Western blot) cannot distinguish latent/active infections (Kasifoglu et al., 2018); nucleic acid methods are optimal for cell banks. Multiplex PCR amplifies multiple targets via specific primer pairs, enabling co-infection detection (Burgart et al., 1992). Isothermal Recombinase Polymerase Amplification (RPA, 37 °C–42 °C, 10–30 min) is fast and instrument-free (Piepenburg et al., 2006); multiplex RPA extends this to HHVs (e.g., Jiang et al.’s duplex RPA for bovine herpesvirus 1/BVDV (Jiang et al., 2024)). Combining multiplex RPA with Lateral Flow Assay (LFA), which uses capillary action and colored labels (e.g., colloidal gold) for visual readouts, creates a simple, low-cost system ideal for on-site screening.

In recent years, significant advances have been made in detection technologies, including real-time quantitative PCR, digital PCR, and various isothermal amplification methods such as RPA, many of which have demonstrated improved sensitivity and rapid detection capabilities. Advances in viral detection technologies have introduced novel isothermal amplification strategies, such as fluorescence-based loop-mediated amplification (Xu et al., 2024), as well as alternative sensing platforms including label-free electrochemical immunosensors (Yu et al., 2022), both of which enable sensitive viral detection. In parallel, artificial intelligence-based approaches have been increasingly applied to EBV-associated disease detection and prognosis through radiomics and histopathological analysis (Peng et al., 2019). However, most existing methods focus on single-virus detection or disease-specific applications. Given the frequent co-infection, latent persistence, and potential reactivation of HHVs, there remains a clear need for rapid, multiplexed, and accessible detection platforms capable of simultaneously identifying multiple HHVs.

RPA, first reported in 2006, uses recombinase, polymerases, and probes (exo/fpg/nfo) for high-sensitivity/specificity amplification. Multiplex RPA integrates with CRISPR (e.g., Li et al.’s RPA-CRISPR/Cas12a for ASFV/GTPV (Xiong et al., 2022)), microfluidics, and AI to boost throughput/portability, finding use in pathogen detection (e.g., HPV typing (Liu et al., 2024)) and food safety (Liu et al., 2021). LFA relies on capillary flow and antigen-antibody/nucleic acid binding (Omidfar et al., 2023); multiplex LFA uses distinct labels/capture zones for simultaneous multi-target detection, suiting point-of-care (POCT) and resource-limited settings (Gomez-Martinez et al., 2018).

**FIGURE 1:**
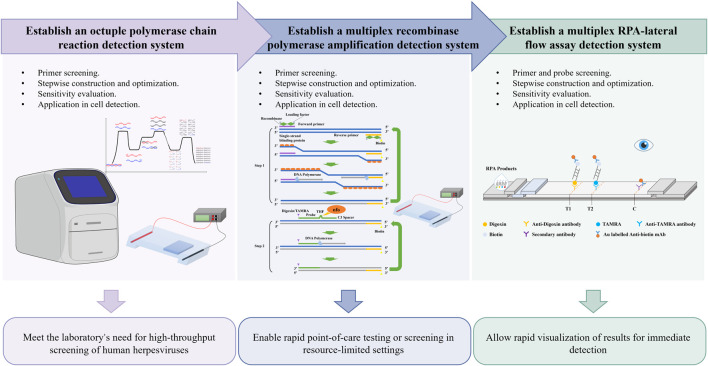
The experimental workflow of rapid multiplex human herpesvirus detection.

This study aims to establish three detection systems to achieve precise and rapid screening of HHVs in cell banks. The experimental workflow is shown in Figure 1. First, a high-specificity and high-sensitivity multiplex PCR system was developed by optimizing primer combinations and reaction conditions. This provides a standardized solution for high-throughput screening of HHVs in laboratory settings. Second, a multiplex RPA system was developed. This system does not require complex instruments and allows rapid isothermal amplification. It addresses the limitations of traditional PCR, which depends heavily on equipment and takes a long time. Multiplex RPA is suitable for preliminary screening in POCT or resource-limited settings. Finally, RPA was combined with LFA to construct a multiplex RPA-LFA system. This system allows rapid visualization of results and enables immediate detection.

## 2 Materials and methods

### 2.1 Cells and viruses

The cells and viruses (HSV-1, HSV-2, VZV, EBV, and HCMV) used in this study were provided by China Center for Type Culture Collection (CCTCC, Wuhan, China). The list of cells is shown in Supplementary Table S1.

### 2.2 Nucleic acid extraction

#### 2.2.1 Cell DNA extraction

Cells cultured in T25 flasks under good growth conditions were digested, centrifuged, and resuspended. DNA was extracted using a blood/cell/tissue genomic DNA extraction kit (Tiangen Biotech, Beijing, China) according to the manufacturer’s instructions. The DNA concentration was measured with a NanoDrop 2000 (Thermo Fisher, Waltham, MA, USA) and diluted to a working concentration of 50 ng/μL with ddH_2_O.

#### 2.2.2 Viral DNA extraction

Cells infected with viruses were cultured in a CO_2_ incubator. Viral DNA was extracted 1 week after the cells became nearly confluent. The culture flasks were freeze-thawed three times at −80 °C and 37 °C. The culture supernatant was collected and centrifuged at 12,000 rpm for 10 min. The supernatant was then filtered through a 0.45 µm sterile filter to obtain virus-containing fluid, which was stored at −80 °C. Viral DNA was extracted using the OMEGA Viral DNA Kit (Omega Bio-tek, Norcross, GA, USA). DNA concentration and purity were measured with a NanoDrop 2000 (Thermo Fisher Scientific, Waltham, MA, USA).

### 2.3 Construction of standard virus plasmids

**FIGURE 2:**
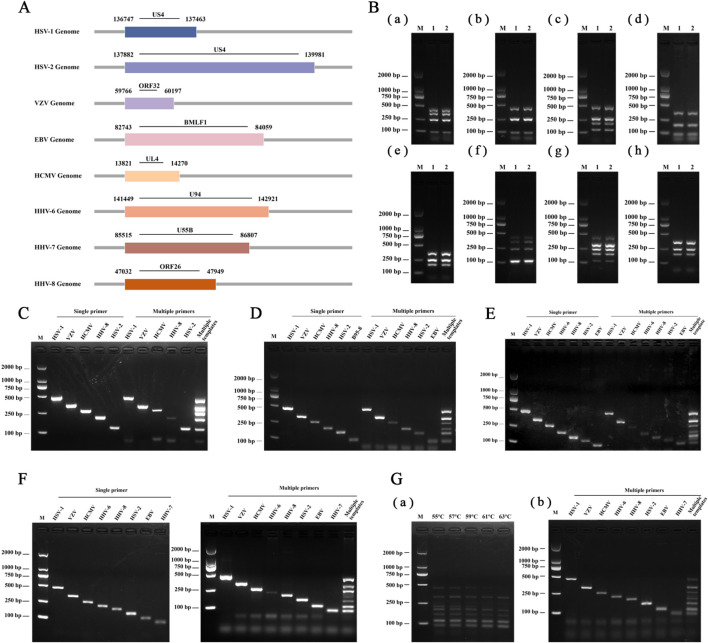
Establishment and optimization of the octuple PCR detection system. **(A)** Conserved fragments used for primer design of eight viruses. **(B)** Eight combinations of the quintuple PCR system. Lanes 1–2: parallel groups. M: DL2000 marker. Combinations: (a) HHV-8 (437 bp) + HSV-1 (367 bp) + HCMV (257 bp) + HHV-6 (142 bp) + EBV (95 bp); (b) HHV-8 (437 bp) + HSV-1 (367 bp) + HCMV (257 bp) + HSV-2 (159 bp) + EBV (95 bp); (c) HSV-1 (453 bp) + HHV-6 (334 bp) + HCMV (257 bp) + HHV-8 (198 bp) + HSV-2 (124 bp); (d) HSV-1 (367 bp) + HSV-2 (290 bp) + HHV-8 (198 bp) + HHV-6 (142 bp) + EBV (95 bp); (e) HSV-2 (439 bp) + HSV-1 (367 bp) + HCMV (257 bp) + HHV-8 (198 bp) + HHV-6 (142 bp); (f) HHV-8 (437 bp) + HSV-1 (367 bp) + HCMV (257 bp) + VZV (122 bp) + EBV (95 bp); (g) HSV-1 (453 bp) + VZV (326 bp) + HCMV (257 bp) + HHV-8 (198 bp) + HSV-2 (124 bp); (h) HSV-2 (439 bp) + HSV-1 (367 bp) + HCMV (257 bp) + HHV-8 (198 bp) + VZV (122 bp). **(C–F)** Stepwise establishment of quintuple, sextuple, septuple, and octuple PCR systems. Each includes: single primer (only specific primers), multiple template (mixture of 5, 6, 7, or 8 templates), and multiple primers (mixture of 5, 6, 7, or 8 primers). **(G)** Optimization of the octuple system. (a) Annealing temperature. (b) Octuple PCR detection system after primer concentration adjustment. The primer concentration of VZV was 150 nM, HSV-1 and EBV were 300 nM, and the other five viruses were 200 nM.

The gene fragments of each virus were synthesized using primers and inserted into the pMD18-T vector (TaKaRa D1010A, Takara Bio, Dalian, China). Conserved gene sequences were selected for primer design, including the US4 gene of HSV-1 (Arvin et al., 2007), the US4 gene of HSV-2 (Kropp et al., 2020), the ORF32 gene of VZV (Cohen et al., 2010), the BMLF1 gene of EBV (Sun et al., 2024), the UL4 gene of HCMV (Arvin et al., 2007), the U94 gene of HHV-6 (Arvin et al., 2007), the U55 B gene of HHV-7 (Black and Pellett, 1999), and the ORF26 gene of HHV-8 (Arvin et al., 2007). The primer sequences and target genes are listed in Table 1 and shown in Figure 2A. In addition, the plasmid containing the HHV-7 gene sequence (NC_001716.2) and all primers designed in this study were synthesized by Tianyi Huiyuan Biotechnology Co., Ltd. (Wuhan, China).

**TABLE 1:** Primer sequences used for plasmid construction.

Virus	GenBank	Primer	Sequence (5′–3′)
HSV-1	NC_001806.2	1-F 1-R	CCTGGTCATCCTTTGCCA TGCTTCGTTATAGCCGTAGT
HSV-2	NC_001798.2	2-F 2-R	GTGACGTACTACCGGCTCAC ATTTACGAGAGCGTACCGGG
VZV	NC_001348.1	3-F 3-R	ATGGAATCGTCTAACATTAACGCG TTAATCGGTGTCAGAATCTTCATCC
EBV	NC_001847.1	4-F 4-R	CTTGGAGACAGGCTTAACCAGACTCA CCATGGCTGCACCGATGAAAGTTAT
HCMV	NC_006273.2	5-F 5-R	TTAGAACGTGGATATCATTACCGATGG TTAGGTCACGGTCAGGTTGTAATAA
HHV-6	NC_001664.4	6-F 6-R	GATGATTTCTGGACTAAGGACAAAT AACAATTTATAGAACGGTTCCTGGC
HHV-8	NC_009333.1	8-F 8-R	ATGGCACTCGACAAGAGTATAGTGG TTAGCGTGGGGAATACCAACAGGAG

The recombinant plasmids were extracted using a TIAN Pure Midi Plasmid Kit (TIANGEN Biotech, Beijing, China). The plasmid concentration was measured with a NanoDrop microvolume spectrophotometer (Thermo Fisher Scientific Inc., Waltham, MA, USA). The DNA copy number of the recombinant EBV plasmid was calculated using the following equation: DNA copy number (copies/µL)=concentration (ng/µL)×10^-9^×6.022×10^23^ (copies/mol)/ [clone size (bp)×660 (g/mol/bp)]. Then, the DNA was serially diluted 10-fold from 1.0 × 10^10^ to 1.0 × 10^0^ copies/µL.

### 2.4 Development of the PCR detection system

#### 2.4.1 Primer screening

The selected genes of each virus were analyzed by BLAST in NCBI to ensure they were highly conserved. Using DNAMAN 8.0 software, 50 primer pairs were designed. After experimental validation, 33 primer pairs with high specificity were selected (sequences are listed in Supplementary Table S2).

The amplification system contained 10 µL of TaKaRa Ex Premier DNA Polymerase Dye Plus (2X), 1 µL of forward primer (10 µM), 1 µL of reverse primer (10 µM), 1 µL of template (50 ng/μL), and ddH_2_O to a total volume of 20 µL. The PCR conditions were: initial denaturation at 94 °C for 1 min; 30 cycles of denaturation at 98 °C for 10 s, annealing at 56 °C for 15 s, and extension at 68 °C for 60 s; followed by a final extension at 72 °C for 5 min. The resulting PCR products were analyzed by 2% agarose gel electrophoresis (120 V, 200 mA).

#### 2.4.2 Basic multiplex PCR

Primer pairs chosen for five, six, seven, or eight viruses were combined in equal proportions. During selection, the target fragment sizes were considered to ensure each fragment could be clearly separated. The multiplex PCR system was based on the PCR system described in 2.4.1, with stepwise increases in the amount of template (1 µL for each template) and primers. Detailed reaction systems are listed in Supplementary Table S3. The resulting PCR products were analyzed by 2% agarose gel electrophoresis (120 V, 200 mA).

#### 2.4.3 Optimization of the reaction conditions

The positive templates of each virus were recombinant plasmids at a concentration of 1 × 10^5^ copies/µL. First, the annealing temperature was optimized. Based on the melting temperatures of the eight selected primer pairs, five annealing temperatures were tested: 55 °C, 57 °C, 59 °C, 61 °C, and 63 °C. The specificity and amplification efficiency of the primers at different temperatures were observed.

Next, the primer concentrations in the octuple PCR system were optimized. The concentrations of the eight primer pairs were adjusted according to previous experimental results. The PCR products were analyzed by agarose gel electrophoresis (120 V, 200 mA) to evaluate the effect of different primer concentrations.

### 2.5 Development of the RPA detection system

#### 2.5.1 Quadruplex RPA

According to the manuals of the TwistAmp® Basic Kit and GenDx ERA Kit, two quadruplex RPA reaction systems (A and B) were prepared (see Supplementary Table S4). First, buffer, primers, template, and ddH_2_O were added to the reaction tube. The reaction was initiated by adding the activator. After mixing thoroughly, the reaction was performed in a 39 °C metal bath for 20 min. The resulting RPA products were analyzed by 2% agarose gel electrophoresis (120 V, 200 mA). The primer sequences for groups A and B are listed in Table 2.

**TABLE 2:** Quadruple RPA primer sequences.

Group	Viruses	Primer	Sequence (5′–3′)	Target fragment
A	HSV-1	1-FP 1-RP	TGGTTCTTGTCGGTGTATCGG GTCGTCCCTTTGAGGTGAGT	453 bp
	HSV-2	2-FP 2-RP	GACGTACTACCGGCTCACCCGCGCTT TACGTGTACGTGTACGACCCGCACGTCTTT	117 bp
	VZV	3-FP 3-RP	ACAGCCAATACGACTACTTAAACAC TAATCGGTGTCAGAATCTTCATCCC	326 bp
	EBV	4-FP 4-RP	CTTGGAGACAGGCTTAACCAGACTCA CCATGGCTGCACCGATGAAAGTTAT	265 bp
B	HCMV	5-FP 5-RP	ACGTGTTACTGGCGGAGTCG TTGAGTGTGGCCAGACTGAG	257 bp
	HHV-6	6-FP 6-RP	TAG​ATG​ATT​GGT​GCA​CGT​ATG​C CTAATGTCTCTTCGTATCCACG	334 bp
	HHV-7	7-FP 7-RP	CTCTGCGATCTATCACTAGTCACTT TAATGCTAATGCTCTCTACGTTCCA	114 bp
	HHV-8	8-FP 8-RP	ACGCTATTCTGCAGCAGCTGTTGGTGTACC GCCAAAGTCTCTGCCGAGTAGGCATACACG	454 bp

#### 2.5.2 Optimization of the RPA reaction conditions

In the quadruplex RPA system, the total volume of mixed primers was fixed at 4 µL. For group A, four different primer ratios were tested, and for group B, three different ratios were tested to optimize primer concentrations. After determining the optimal ratio, eight experiments were performed to optimize the total volume of mixed primers. Next, the reaction time was fixed at 20 min, and the reaction temperature was optimized using six temperature gradients: 35 °C, 37 °C, 39 °C, 41 °C, 43 °C, and 45 °C. Then, the reaction time was optimized at a fixed temperature of 41 °C with amplification times of 5, 10, 15, 20, 25, and 30 min. Finally, the amount of activator was optimized. The best reaction conditions were determined by observing the agarose gel electrophoresis results (120 V, 200 mA).

### 2.6 Development of the RPA-LFA detection system

#### 2.6.1 Basic duplex RPA-LFA

1. Before introducing the probe: The forward primer was labeled with TAMRA or Digoxin at the 5′end, and the reverse primer was labeled with Biotin at the 5′end. HHV-6 and HCMV templates and primers were added in equal proportions. The remaining reagents were the same as those used in the basic RPA system. The amplification product was diluted 1:50 with diluent, and 50 µL was added dropwise to the spotting area of the lateral flow dipstick. The results were observed after 3 min.
2. After introducing the probe: The forward primer was not labeled, and the reverse primer was labeled with Biotin. A TAMRA or Digoxin-labeled probe (0.6 µL), 1.5 µL of nfo enzyme, and 5 µL of nfo buffer were added to the reaction system. The remaining reagents were the same as those used in the RPA system (nfo enzyme and nfo buffer were from FastDigest, Inc., Waltham, MA, USA). The amplification product was diluted 1:50 with diluent, and 50 µL was added dropwise to the spotting area of the lateral flow dipstick. The results were observed after 3 min.

The primers and probes are listed in Table 3.

**TABLE 3:** Primer and probe sequences for RPA-LFA.

Virus	Primer/Probe	Sequence (5′–3′)
HHV-6	6-RPA-FP1	[TAMRA]TAGATGATTGGTGCACGTATGC
	6-RPA-FP2	TAGATGATTGGTGCACGTATGC
	6-RPA-RP	[BIOTIN]CTAATGTCTCTTCGTATCCACG
	6-Probe	[TAMRA]GTCTTCAGTCTAAGTTACATCCCCTTTGCCC [THF]TCTTCAAATTATTAG [C3-SPACER]
HCMV	5-RPA-FP1	[DIGOXIN]GAG​GTC​TTC​AGA​ACA​AAA​CGG​AAG​A
	5-RPA-FP2	GAGGTCTTCAGAACAAAACGGAAGA
	5-RPA-RP	[BIOTIN]CACGGTCAGGTTGTAACAAGAGTAATA
	5-Probe	[DIGOXIN]CGGAAAACAGCGGAAAGTATTATTTCAAACG [THF]GAAGATGTGAATTCC [C3-SPACER]

#### 2.6.2 Optimization of the duplex RPA-LFA system

The duplex RPA-LFA system was optimized based on primer ratio and total volume, probe ratio and total volume, reaction temperature, and reaction time. Three experiments were conducted to optimize primer ratios, with HHV-6 and HCMV primers mixed at 1:1, 2:1, and 3:1. Next, the total volume of the mixed probes was adjusted. The probe ratios were then optimized at 1:1, 2:1, and 3:1. The total amount of mixed primers was also adjusted. The optimal reaction temperature was tested using five gradients: 35 °C, 37 °C, 39 °C, 41 °C, and 43 °C. Reaction time was optimized by performing amplification for 5, 10, 15, and 20 min at a fixed temperature. The amplification product was diluted 1:50 with diluent, and 50 µL was added dropwise to the sample area of the lateral flow dipstick. Results were observed after 3 min.

### 2.7 Comparative evaluation of the detection systems

This section describes the evaluation of three detection systems developed in this study: the octuple PCR system, the two quadruplex RPA systems, and the duplex RPA-LFA system. Sensitivity and specificity were assessed under standardized conditions to ensure comparable analytical performance across platforms. All experiments were performed in at least three independent replicates.

#### 2.7.1 Sensitivity evaluation

For both the octuple PCR system and the two quadruplex RPA systems, the viral plasmids were serially diluted to 1 × 10^7^, 1 × 10^6^, 1 × 10^5^, 1 × 10^4^, 1 × 10^3^, 1 × 10^2^, 1 × 10^1^, and 1 × 10^0^ copies/µL. For mixed-virus detection, 1 µL of each plasmid at each dilution was used as the template (8 µL total for PCR and 4 µL total for RPA). For single-virus detection, 1 µL of each diluted plasmid was used together with the mixed primer set. All PCR and RPA amplification products were analyzed by agarose gel electrophoresis under identical conditions (120 V and 200 mA).

For the duplex RPA-LFA system, HHV-6 and HCMV plasmids were diluted to 1 × 10^8^, 1 × 10^7^, 1 × 10^6^, 1 × 10^5^, 1 × 10^4^, 1 × 10^3^, 1 × 10^2^, and 1 × 10^1^ copies/µL. For simultaneous detection, 1 µL of each diluted plasmid (2 µL total) was used as the template. For single-virus detection, 1 µL of the corresponding plasmid at each dilution was used. After amplification, product was diluted 1:50 with diluent, and 50 µL was added dropwise to the sample area of the lateral flow dipstick. Results were observed after 3 min.

#### 2.7.2 Specificity evaluation

All PCR and RPA amplification products were analyzed by agarose gel electrophoresis under the same electrophoresis conditions (120 V, 200 mA). The templates and testing strategies differed among systems. For the octuple PCR system, DNA from three cell lines was used as the template: B95-8 (EBV-positive), BCBL-1 (HHV-8-positive), and HUV-EC-C (HHV-6-positive). Amplification was performed with both single-virus primers and the mixed primer set at 59 °C. For the quadruplex RPA systems, group A templates included HSV-1, HSV-2, VZV DNA, and DNA from B95-8 (EBV-positive) cells. Group B templates included HHV-7 plasmid, HCMV DNA, and DNA from BCBL-1 (HHV-8-positive) and HUV-EC-C (HHV-6-positive) cells. Single and mixed templates were amplified under optimal conditions. To further assess cross-reactivity, group A primers were tested with the four positive templates of group B, and group B primers were tested with the four positive templates of group A.

For the duplex RPA-LFA system, ten previously confirmed negative cell samples, HCMV viral DNA, and HUV-EC-C DNA (HHV-6-positive) were tested. Test lines (T1 for HCMV and T2 for HHV-6) and control lines (C) were recorded to determine specificity.

## 3 Results

### 3.1 Establishment and optimization of the octuple PCR detection system

In this study, primers were designed and screened based on conserved fragments of eight human herpesviruses (HHVs) (Figure 2A). After individual validation, a total of 33 primer pairs with high specificity were selected (Supplementary Table S2). To ensure that primers did not interfere with each other and that the amplified fragment sizes were suitable, primers for five different viruses were first selected in different combinations. DNA extracted from viral cultures was used as the positive template for PCR amplification. The PCR products were analyzed by 3% agarose gel electrophoresis (120 V, 220 A) to attempt the construction of a quintuple PCR detection system. The results are shown in Figure 2B. Among eight experimental groups, only the group using HSV-1, VZV, HCMV, HHV-8, and HSV-2 as templates, with target fragment sizes of 453 bp, 326 bp, 257 bp, 198 bp, and 124 bp, respectively (group g), successfully amplified all five target fragments. Therefore, this combination was chosen for subsequent experiments.

To further confirm the specificity of this primer set, each primer pair was individually added to its corresponding positive template for single PCR amplification. At the same time, the five primer pairs were mixed in equal proportions to form a mixed primer set. PCR was performed with either the five individual positive templates or the mixture of all five positive templates. The results of agarose gel electrophoresis are shown in Figure 2C. The amplified fragments of all five viruses matched the expected sizes and showed high specificity. In the mixed primer set, even if only one positive template was present, it could be accurately detected with specificity. When all five positive templates were present simultaneously, the amplified fragments matched the expected sizes. The quintuple PCR detection system was preliminarily established.

Based on these previous experiments, primers for detecting EBV, HHV-6, and HHV-7 were gradually added to the system. Hexaplex, heptaplex, and octaplex PCR detection systems for human herpesviruses were successfully established (Figures 2D–F). Notably, in the hexaplex system, the EBV primer amplified a fragment of 95 bp. Later, to avoid overlap with the HHV-7 fragment in the octaplex system, the EBV fragment size was adjusted to 85 bp.

To further improve the detection performance of this system, the positive templates for each virus were replaced with the corresponding viral plasmid standards. The reaction conditions were optimized by adjusting annealing temperature and primer concentration. When the primer ratios were unchanged, the results of gel electrophoresis at different annealing temperatures are shown in Figure 2Ga. The amplification of VZV, HHV-8, HSV-2, HHV-7, and EBV remained stable across the tested temperature range. However, the target bands of HSV-1 and HHV-6 gradually weakened as the temperature increased, while the HCMV target band became clearer with higher temperature. Considering the amplification performance of all eight target fragments, 59 °C was selected as the optimal annealing temperature.

At an annealing temperature of 59 °C, primer concentrations were further optimized. The resulting system showed bright and specific amplification bands, as shown in Figure 2Gb. The optimal primer concentrations in this system were as follows: VZV primers at 150 nM, HSV-1 and EBV primers at 300 nM, and the remaining five virus primers at 200 nM each. With these conditions, the octuple PCR detection system for human herpesviruses was successfully established and optimized.

### 3.2 Sensitivity and specificity of the octuple PCR detection system

**FIGURE 3:**
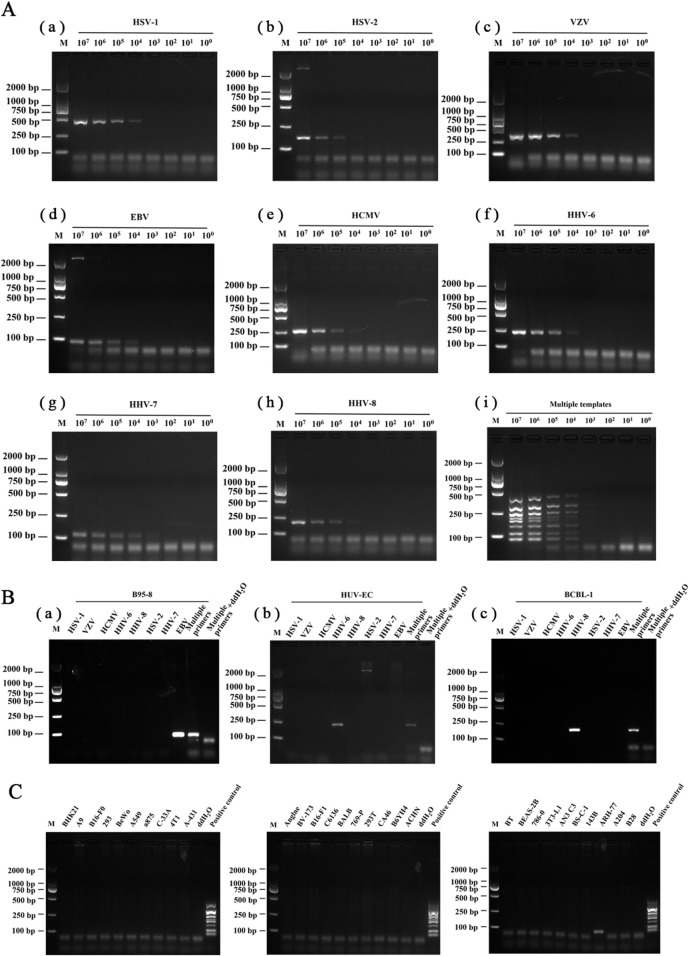
Sensitivity and specificity of the octuple PCR detection system. **(A)** Sensitivity of the octuple PCR system for detecting 8 herpesviruses. (a–h) Correspond to the individual herpesvirus templates for HSV-1, HSV-2, VZV, EBV, HCMV, HHV-6, HHV-7, and HHV-8, respectively; **(i)** Multiple templates. **(B)** Specificity of the octuple PCR system. (a–c) B95-8 (EBV-positive), BCBL-1 (HHV-8-positive), and HUV-EC-C (HHV-6-positive) laboratory HHV-positive cell lines were used as DNA templates, respectively. Detection was performed using single-primer (HSV-1, HSV-2, VZV, EBV, HCMV, HHV-6, HHV-7, and HHV-8) and multiplex primer sets. **(C)** Application of the octuple PCR system in cells. M: DL2000 marker; Multiple template: mixture of 8 templates; Multiple primers: mixture of 8 primers.

After successfully constructing the octuple PCR detection system, we next evaluated its analytical performance, focusing on sensitivity and specificity. The viral plasmids were diluted to concentrations of 1 × 10^7^, 1 × 10^6^, 1 × 10^5^, 1 × 10^4^, 1 × 10^3^, 1 × 10^2^, 1 × 10^1^, and 1 × 10° copies/μL. For each dilution, 1 μL of each plasmid (a total of 8 μL) was used as template and added to the mixed primer system for amplification under optimal conditions. The results of agarose gel electrophoresis are shown in Figure 3Ai. Clear bands were observed when plasmids were diluted to 1 × 10^4^ copies or higher, but no clear bands were observed at 1 × 10^3^ copies or lower. Therefore, the lower detection limit of the octuple PCR for simultaneous detection of eight herpesviruses was 1 × 10^4^ copies per 50 μL reaction.

Although the system was confirmed to detect all eight HHVs simultaneously, many cultured cells may be infected with only one or a few viruses. Therefore, the sensitivity for detecting a single herpesvirus was also tested. For this, 1 μL of each plasmid at eight concentration gradients was individually added to the mixed primer system for amplification. The agarose gel results are shown in Figures 3Aa–h. For HSV-1, VZV, HCMV, HHV-6, HHV-8, HSV-2, and EBV, a plasmid copy number of ≥1 × 10^4^ could be detected individually. At 1 × 10^3^ copies or lower, no clear bands were observed. For HHV-7, bands were still visible at 1 × 10^3^ copies. This discrepancy may be attributed to differences in primer binding efficiency, amplicon length, GC content, or local secondary structure of the target regions, all of which can influence amplification efficiency in multiplex PCR systems. Therefore, the lower detection limit for HHV-7 was 1 × 10^3^ copies, while for the other seven viruses it was 1 × 10^4^ copies.

To further validate the specificity of this system, three laboratory HHV-positive cell lines were selected as DNA templates: B95-8 (EBV-positive), BCBL-1 (HHV-8-positive), and HUV-EC-C (HHV-6-positive). PCR was performed using either the single-virus primers or the mixed primer set. The results are shown in Figure 3B. Both single primers and mixed primers produced specific amplification, confirming the specificity of the system.

After establishing the octuple PCR system and determining its sensitivity, the system was applied to detect HHVs in 30 cell lines preserved at the China Center for Type Culture Collection (CCTCC) to assess practical performance. The results are shown in Figure 3C. Among the 30 cell lines, ARH-77 showed a specific EBV-positive band, consistent with published data. No specific herpesvirus bands were detected in the other 29 cell lines, which matched expectations.

### 3.3 Establishment of the quadruple RPA detection system

Although the octuple PCR system achieved reliable results, it still required thermal cycling equipment and longer reaction times. Therefore, we aimed to establish a faster, isothermal alternative based on RPA. We initially tried to use the primer sets from the multiplex PCR system to build a corresponding octuple RPA detection system. Five experimental groups with different primer concentrations were set up to detect all eight viruses. However, agarose gel electrophoresis showed that the number of amplified bands was limited, with a maximum of only four bands (Supplementary Figure S1). A literature search revealed no prior reports of an octuple RPA detection system.

**FIGURE 4:**
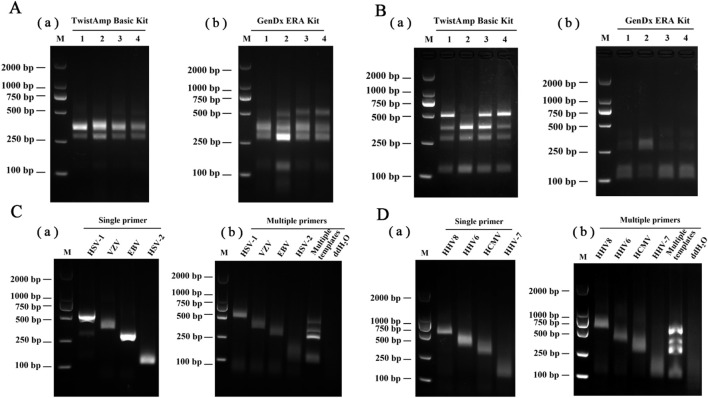
Establishment of quadruple RPA detection systems. **(A)** Optimization of primer concentrations in Group A (HSV-1, VZV, EBV, HSV-2). (a) Using TwistAmp® Basic kit. (b) Using GenDx kit. 1-4 represents different combinations: 1) all 200 nM; 2) 500/200/500/500 nM; 3) 500/150/200/200 nM; 4) 500/100/200/200 nM; **(B)** Optimization of primer concentrations in Group B (HHV-8, HHV-6, HCMV, HHV-7). (a) Using TwistAmp® Basic kit. (b) Using GenDx kit. 1-4 represents different combinations: 1) all 200 nM; 2) 150/200/200/500 nM; 3) 200/200/200/500 nM; 4) 100/200/200/500 nM. **(C)** Establishment of Group A quadruple RPA detection system. **(D)** Establishment of Group B quadruple RPA detection system. M: DL2000 marker; Multiple template: mixture of 4 templates; Multiple primers: mixture of 4 primers; Single primer: only specific primers.

To ensure system stability, the eight viruses were divided into two groups to construct two quadruple RPA detection systems: Group A for detecting HSV-1, HSV-2, VZV, and EBV (Figure 4A), and Group B for detecting HCMV, HHV-6, HHV-7, and HHV-8. Because primers suitable for multiplex RPA differ from those used in multiplex PCR, and dedicated design software is lacking, some primers were replaced during experiments.

Initially, the commonly used TwistAmp® Basic kit was selected to directly establish the quadruple RPA system. After multiple attempts, the Group A system could not be successfully constructed. Considering the influence of the kit, another kit from GenDx was introduced for comparison, and four experimental groups with different primer concentrations were set up. Using the TwistAmp® Basic kit, only VZV and EBV target fragments were successfully amplified, while HSV-1 and HSV-2 bands were almost undetectable (Figure 4Aa). In contrast, using the GenDx kit, all four target fragments were amplified in all four experimental groups, although band brightness varied. In the second experimental group, all four bands were clear, and the EBV band was the brightest (Figure 4Ab). Further testing with different primers and templates using the GenDx kit showed consistent amplification results (Figure 4C). Therefore, the Group A quadruple RPA system was established using the GenDx kit.

Similarly, amplification performance for Group B viruses was tested with different kits. Interestingly, using the GenDx isothermal amplification kit, only EBV and HHV-7 bands were amplified, and the other two bands were weak. In contrast, the TwistAmp® Basic kit amplified all four targets in all four experimental groups (Figure 4B). Further validation with different templates and primers confirmed the expected results. Therefore, the TwistAmp® Basic kit was used for subsequent optimization of the Group B quadruple RPA detection system. Further testing with different primers and templates showed consistent amplification results (Figure 4D).

### 3.4 Optimization of the quadruplex RPA detection system

**FIGURE 5:**
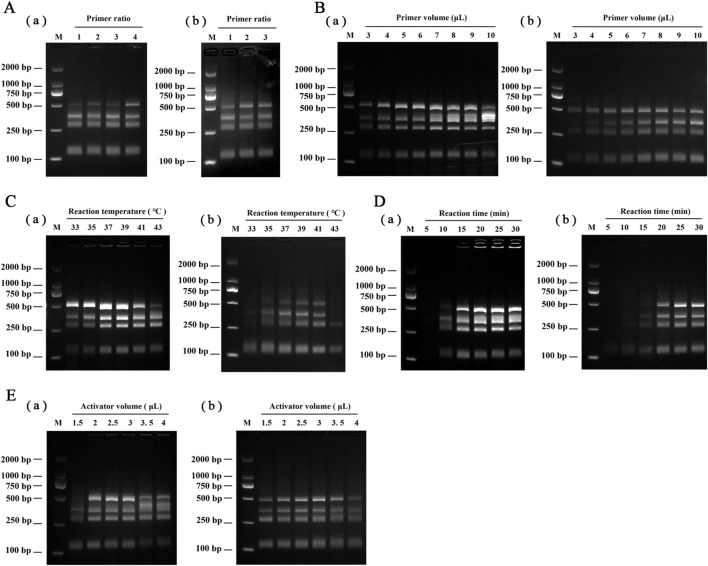
Optimization of the quadruple RPA detection systems. **(A)** Primer ratio optimization. (a) Group A: HSV-1/VZV/EBV/HSV-2 primer concentrations tested as 1) 500/150/150/400 nM; 2) 540/200/150/500 nM; 3) 540/150/150/400 nM; 4) 600/150/150/400 nM. (b) Group B: HHV-8/HHV-6/HCMV/HHV-7 primer concentrations tested as 1) 200/200/200/200 nM; 2) 150/200/200/200 nM; 3) 100/180/200/200 nM. **(B)** Optimization of total primer volume (from 3 to 10 μL). (a) Group A; (b) Group B. **(C)** Optimization of reaction temperature (from 33 °C to 43 °C). (a) Group A; (b) Group B. **(D)** Optimization of reaction time (from 5 to 30 min). (a) Group A; (b) Group B. **(E)** Optimization of activator dosage (from 1.5 to 4 μL). (a) Group A; (b) Group B. M: DL2000 marker.

After the initial establishment of two quadruple RPA systems, further optimization was required to improve amplification efficiency, balance signal intensity, and minimize non-specific reactions. This optimization process focused on fine-tuning primer ratios, total primer amounts, temperature, reaction time, and activator concentration to achieve uniform and stable amplification. First, the total primer volume was fixed at 4 μL, and different primer ratios were tested in both groups. Figure 5A shows that for the A group quadruplex RPA system, experiment 4 had the best amplification. Under these conditions, the primer ratio of HSV-1, VZV, EBV, and HSV-2 was 10:3:3:4, which was determined as the optimal primer ratio. For the B group system, three adjusted experimental groups produced fairly even amplification of all four targets, and experiment 3 showed the best result. The optimal primer ratio for the B group was set as HHV-8: HHV-6: HCMV: HHV-7 = 5: 9: 10: 10.

Next, the primers were mixed at the optimal ratios, and the total primer volume was optimized using eight experimental groups. For A group, as shown in Figure 5Ba, when the total primer volume increased to 7 μL, non-specific amplification of VZV appeared. The best amplification was observed at 6 μL total primer volume. The final primer concentrations in the A group were 900 nM (HSV-1), 225 nM (VZV), 225 nM (EBV), and 600 nM (HSV-2). For the B group, increasing the primer volume did not cause strong non-specific amplification. Instead, the target bands became brighter. At 8 μL total primer volume, the target bands were clear, and the final primer concentrations were 200 nM (HHV-8), 360 nM (HHV-6), 400 nM (HCMV), and 400 nM (HHV-7).

To determine the optimal reaction temperature, six gradients were tested: 33, 35, 37, 39, 41, and 43 °C. Both groups showed that 41 °C gave clear and even target bands, while higher temperatures reduced amplification efficiency (Figure 5C). Therefore, 41 °C was selected as the optimal temperature.

To optimize reaction time, six time points were tested: 5, 10, 15, 20, 25, and 30 min. Gel electrophoresis showed that the target bands became clearer with longer reaction time (Figure 5D). For the A group and B group, target bands were sufficiently clear at 15 min and 20 min, respectively.

The activator amount is also critical in RPA. Six gradients (1.5, 2, 2.5, 3, 3.5, 4 μL) were tested. The best results were observed at 2–3 μL (Figure 5E). Higher amounts caused diffused bands. Therefore, the optimal activator volume was set as 2 μL for the A group and 2.5 μL for the B group.

In conclusion, after optimizing primer concentration, reaction temperature, reaction time, and activator volume, the final conditions were: A group: HSV-1 900 nM, VZV 225 nM, EBV 225 nM, HSV-2 600 nM; 41 °C; 15 min; activator 2 μL; B group: HHV-8 200 nM, HHV-6 360 nM, HCMV 400 nM, HHV-7 400 nM; 41 °C; 20 min; activator 2.5 μL.

### 3.5 Sensitivity of the quadruplex RPA detection system

Following optimization, the sensitivity of the two quadruple RPA systems was examined to determine their detection limits under optimal reaction conditions. Similar to the octaplex PCR system, we tested the sensitivity of the quadruplex RPA system for simultaneously detecting four human herpesviruses under the optimized reaction conditions. Viral plasmids were diluted to 1 × 10^7^, 1 × 10^6^, 1 × 10^5^, 1 × 10^4^, 1 × 10^3^, 1 × 10^2^, 1 × 10^1^, and 1 × 10° copies/μL. For each concentration, 1 μL of each plasmid was added to the corresponding reaction system for amplification, and the products were analyzed by gel electrophoresis.

**FIGURE 6:**
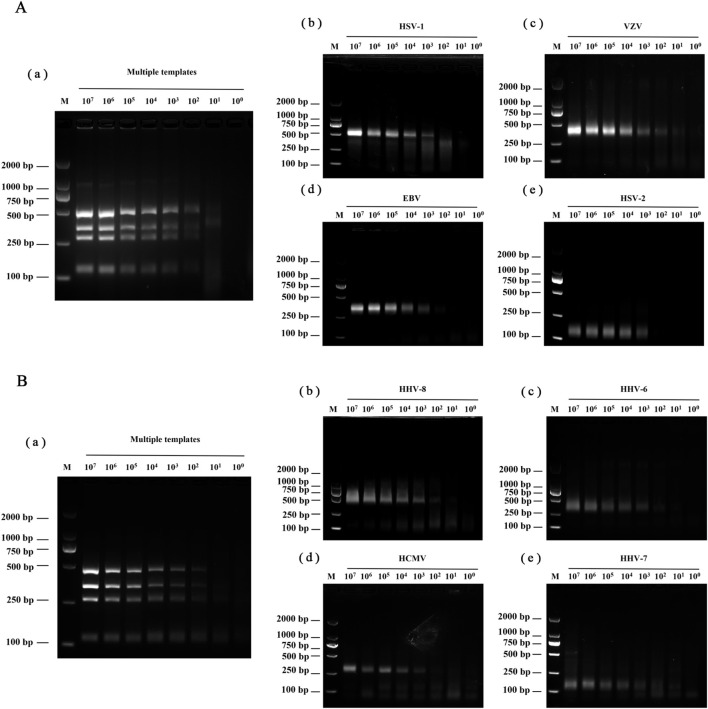
Detection sensitivity of the quadruple RPA systems. **(A)** Sensitivity test of the Group A quadruple RPA system for four herpesviruses. **(B)** Sensitivity test of the Group B quadruple RPA system for four herpesviruses. M: DL2000 marker; Multiple template: mixture of 4 templates.

As shown in Figure 6Aa and Figure 6Ba, both A and B group quadruplex RPA systems could simultaneously detect four human herpesviruses with a minimum detection limit of 1 × 10^3^ copies (50 μL system), which is more sensitive than the octaplex PCR system.

We also tested the sensitivity for detecting single viruses in the A and B group quadruplex RPA systems. As shown in Figures 6Ab–e,Bb–e, the minimum detection limit for individual viruses was 1 × 10^3^ copies (50 μL system). Among them, VZV showed the highest sensitivity, with a minimum detection limit of 1 × 10^2^ copies (50 μL system).

Compared with the octuple PCR detection system, the quadruplex RPA system achieved a tenfold increase in sensitivity, providing a solid foundation for the subsequent development of the RPA-LFA visual detection platform.

### 3.6 Specificity of the quadruplex RPA detection system

To ensure reliable detection, we tested the specificity of the A and B group quadruplex RPA systems. Unlike the previous experiments, where plasmid standards were used as templates, we used DNA extracted from live viruses to evaluate the system’s performance in complex samples.

For the A group system, HSV-1, VZV, and HSV-2 templates were replaced with DNA from their respective live viruses, and the EBV template was replaced with B95-8 cell DNA. For the B-group system, templates were replaced with HHV-7 plasmid, HCMV viral DNA, and DNA from BCBL-1 (HHV-8-positive) and HUV-EC-C (HHV-6-positive) cells. All reactions were performed under the optimized conditions.

**FIGURE 7:**
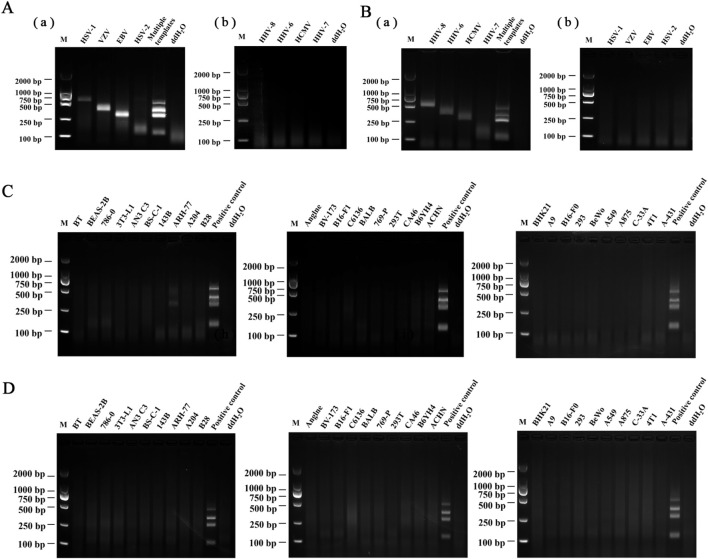
Detection specificity of the quadruple RPA systems. **(A)** Specificity validation of the Group A quadruple RPA system. **(B)** Specificity validation of the Group B quadruple RPA system. **(C)** Detection of cells by the Group A quadruple RPA system. **(D)** Detection of cells by the Group B quadruple RPA system. M: DL2000 marker; Multiple template/Positive control: mixture of 4 positive templates.

As shown in Figures 7Aa,Ba, when the quadruplex RPA system detected a single virus, only one specific target band was amplified. When all four viruses were present, all were detected simultaneously, confirming the specificity of the quadruplex RPA system.

We also evaluated potential cross-reactivity between the A and B group systems. The A-group system was tested with B group viruses, and *vice versa*. As shown in Figures 7Ab,Bb, all results were negative, further confirming the specificity and reliability of both quadruplex RPA systems.

The quadruplex RPA system is valuable for routine screening in cell banks. To verify its effectiveness in real cell samples, we used the A and B group systems to test 30 cell lines previously analyzed by the Octaplex PCR system. As shown in Figures 7C,D, one cell line, ARH-77, tested positive for EBV, while all others were negative, fully consistent with the multiplex PCR results. These results demonstrate that the quadruplex RPA system is efficient, reliable, and suitable for cell bank screening and viral detection applications.

### 3.7 Establishment and optimization of the duplex RPA-LFA detection system

While the RPA systems provided rapid nucleic acid detection, they still required gel electrophoresis for visualization. Therefore, we further combined RPA with LFA technology to achieve rapid, visual, and equipment-free detection. The forward primer for HHV-6 was labeled with TAMRA, and the forward primer for HCMV was labeled with Digoxin, while the reverse primers for both viruses were labeled with Biotin. The corresponding viral templates were added to the labeled primer sets and incubated at 41 °C for 20 min, resulting in amplification of products carrying both labels.

**FIGURE 8:**
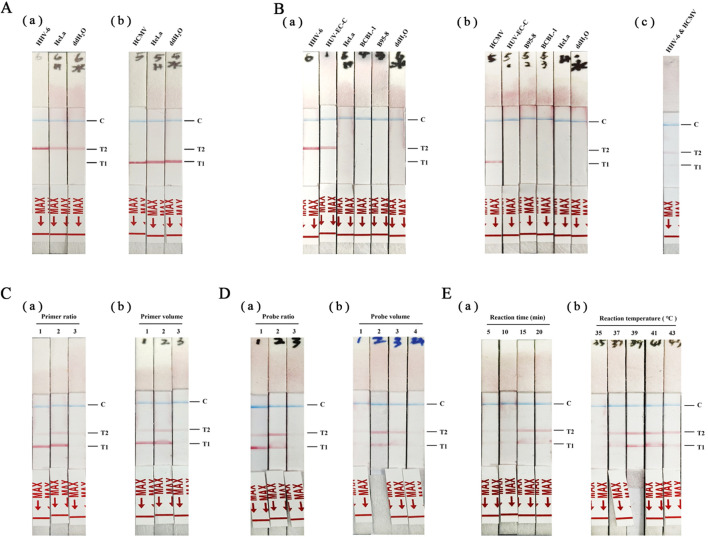
Establishment and optimization of the duplex RPA-LFA system. **(A)** Preliminary assay. (a) HHV-6 single detection. (b) HCMV single detection. **(B)** Probe introduction. (a) HHV-6 with probe. (b) HCMV with probe. (c) Duplex detection with both probes and templates. **(C)** Primer optimization. (a) Primer concentration. 1) both 400 nM. 2) HHV-6 800 nM, HCMV 400 nM. 3) HHV-6 1,200 nM, HCMV 400 nM. (b) Primer volume. 1) 4 μL. 2: 5 μL. 3) 6 μL. **(D)** Probe optimization. (a) Probe concentration. 1) both 60 nM. 2) HHV-6 120 nM, HCMV 60 nM. 3) HHV-6 180 nM, HCMV 60 nM. (b) Probe volume. 1) 0.4 μL. 2) 0.6 μL. 3) 0.8 μL. 4: 1 μL. **(E)** Reaction conditions. (a) Time optimization (from 5 to 20 min). (b) Temperature optimization (from 35 °C to 43 °C). C: Control line. T1: HCMV test line. T2: HHV-6 test line. BCBL-1: HHV-8-positive cells. HUV-EC-C: HHV-6-positive cells. B95-8: EBV-positive cells. HeLa: negative control. Negative and blank controls show only the C line.

Next, 1 μL of the amplification product was mixed with 49 μL of lateral flow dipstick diluent and applied to the strip. After 3 min at room temperature, as shown in Figure 8A, both HHV-6 and HCMV positive samples showed visible test lines, but the negative control and ddH2O blank control also showed false-positive lines. This was likely caused by primer dimers producing false signals on the strips.

To eliminate the false-positive signals, we introduced probes and moved the labels from the forward primers to the probes. A TAMRA-labeled probe was used for HHV-6, and a Digoxin-labeled probe was used for HCMV. As shown in Figure 8B(a,b), the negative and blank controls showed only the control line (C), and false positives disappeared. When both probes were added together, the test lines T1 and T2 showed clear signals for mixed HHV-6 and HCMV plasmids, while negative and blank controls remained negative (Figure 8B(c)). This established the preliminary duplex RPA-LFA detection system.

To further improve detection, we optimized the system for four factors: primer ratio and total amount, probe ratio and total amount, reaction time, and reaction temperature. The optimized conditions (Figures 8C–E) were: total primer volume of 5 μL, final primer concentrations of 1,000 nM for HHV-6 and 500 nM for HCMV, final probe concentrations of 120 nM for HHV-6 and 60 nM for HCMV, reaction time of 15 min, and reaction temperature of 39 °C.

### 3.8 Sensitivity and specificity of the duplex RPA-LFA detection system

**FIGURE 9:**
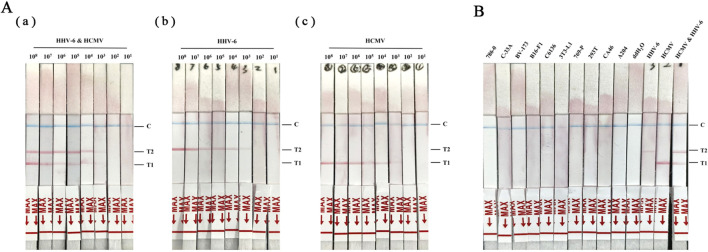
Sensitivity and specificity of the duplex RPA-LFA system. **(A)** Sensitivity of the duplex RPA-LFA system. (a) Amplification with different copy numbers of HHV-6 and HCMV plasmids. (b) Amplification with different copy numbers of HHV-6 plasmid. **(c)** Amplification with different copy numbers of HCMV plasmid. **(B)** Specificity of the duplex RPA-LFA system. c, Control line. T1: HCMV test line. T2: HHV-6 test line.

To evaluate the detection sensitivity of the duplex RPA-LFA system, HHV-6 and HCMV plasmids were serially diluted to concentrations of 1 × 10^8^, 1 × 10^7^, 1 × 10^6^, 1 × 10^5^, 1 × 10^4^, 1 × 10^3^, 1 × 10^2^, and 1 × 10^1^ copies/μL. The detection limits were assessed both for simultaneous detection of the two viruses and for individual detection of HHV-6 and HCMV. As shown in Figure 9A, the duplex RPA-LFA system (50 μL) achieved a minimum detection limit of 1 × 10^3^ copies for both single-virus detection and dual-virus detection. These results are consistent with those obtained using the previously established quadruplex RPA system, indicating that the duplex RPA-LFA system maintains high sensitivity while providing rapid visual results.

To further evaluate specificity, 10 previously tested negative cell samples, HCMV viral DNA, and HUV-EC-C (HHV-6) cell DNA were tested using the duplex RPA-LFA system. After development on the lateral flow strips, as shown in Figure 9B, all negative cell samples showed negative results, consistent with the established octaplex PCR and quadruplex RPA systems. HCMV DNA produced a visible line at T1, and the HHV-6 positive HUV-EC-C cells produced a line at T2, as expected. These results demonstrate that the duplex RPA-LFA system provides rapid visual detection while ensuring accurate and reliable results.

## 4 Discussion

Cell culture is a core technology in biomedical research, and contamination during its handling can severely affect experimental outcomes or related biological products. Human herpesviruses (HHVs) are widely prevalent in the population, with more than 90% of adults having been infected with at least one type of herpesvirus. Currently, eight herpesviruses are known to infect humans, including HSV-1, HSV-2, VZV, EBV, HCMV, HHV-6, HHV-7, and HHV-8. Among them, EBV has a global infection rate exceeding 95%, and HSV-1 infects nearly 70% of the world’s population. Human herpesviruses remain latent in the body after primary infection, potentially leading to blindness, encephalitis, severe neuralgia, and, in some cases, congenital malformations or neonatal death, posing a particularly severe threat to immunocompromised individuals. Therefore, timely detection of human herpesviruses in cell cultures is crucial for ensuring the accuracy of experimental research and safeguarding public health. In addition to applications in cell culture quality control, the clinical relevance of HHV detection, particularly in transplantation medicine, also plays an important role. In immunosuppressed patients, such as those undergoing organ or hematopoietic stem cell transplantation, latent HHVs can be reactivated, leading to severe complications including graft rejection, opportunistic infections, and increased mortality. Notably, co-reactivation of multiple HHVs has been associated with poorer clinical outcomes, highlighting the importance of multiplex detection strategies.

**FIGURE 10:**
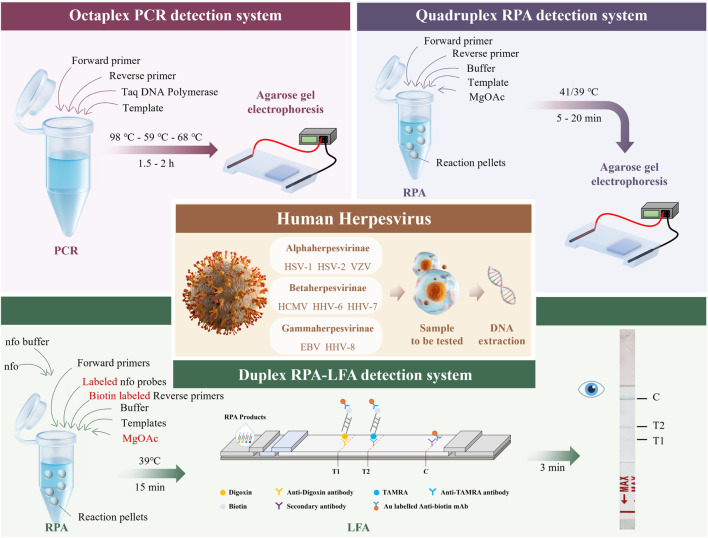
The workflow for three detection systems.

However, existing detection methods for human herpesviruses are often complex, time-consuming, and limited in scope, usually targeting only one or a few types of herpesviruses. This limitation makes them inadequate for simultaneous screening of multiple HHVs in complex samples. To address this gap, we developed three detection systems using conventional PCR technology and emerging recombinase polymerase amplification combined with lateral flow assay (RPA-LFA): the octoplex PCR detection system, the quadruplex RPA detection system, and the duplex RPA-LFA detection system. These systems enable synchronous screening for eight human herpesviruses across different application scenarios. The detailed workflow for these detection systems is shown in Figure 10. The octuple PCR system enables the simultaneous detection of eight human herpesviruses, making it highly suitable for high-throughput laboratory screening, especially when handling complex samples such as mixed infections or large cell-banks that require comprehensive viral assessment. The quadruplex RPA system reduces the reaction time to 15–20 min and shows a tenfold increase in sensitivity compared with the octuple PCR system. These features make it particularly appropriate for rapid screening in routine laboratory workflows, pre-experiment quality control, or situations requiring faster turnaround time but still relying on basic molecular equipment. Moreover, the duplex RPA-LFA system achieves rapid visual detection without the need for specialized instruments. This system is ideal for point-of-care testing, on-site preliminary diagnosis, and resource-limited settings such as community clinics, field investigations, and low-equipment laboratories.

Compared with existing technologies (Table 4), the three detection systems established in this study have certain advantages. Although previous studies have used multiplex PCR to simultaneously detect six human herpesviruses (HSV-1/2, VZV, EBV, HCMV, HHV-6, and HHV-7) (Tanaka et al., 2009), most multiplex detection systems can only detect a few types of herpesviruses. For example, quadruplex PCR has been used to simultaneously detect HSV-1, HSV-2, VZV, and HCMV, which is suitable for diagnosing ocular viral infections (H et al., 2021). The octoplex PCR detection system established in this study can detect all eight human herpesviruses in a single-tube reaction under conventional laboratory conditions, making it suitable for comprehensive screening of samples for herpesvirus infection. Compared with multiplex PCR, multiplex RPA can complete the reaction quickly under isothermal conditions; however, current studies on directly using multiplex RPA to detect multiple human herpesviruses are limited, usually requiring combination with other techniques and typically detecting only a single virus. For example, Shin K. et al. Developed an RPA-CRISPR-based rapid diagnostic platform for HCMV (Shin et al., 2024). The two quadruplex RPA detection systems established in this study, combined with conventional gel electrophoresis, allow rapid and efficient detection of eight human herpesviruses in just two reactions, significantly reducing experimental costs and equipment dependency while simplifying the procedure. Moreover, combining RPA with lateral flow assay (LFA) can greatly shorten detection time. However, most studies using RPA-LFA detect only a single human herpesvirus, such as a rapid EBV detection system previously established in our laboratory (Sun et al., 2024). Therefore, the duplex RPA-LFA detection system developed in this study offers certain advantages by enabling rapid, visual detection of both HCMV and HHV-6 while maintaining operational simplicity.

**TABLE 4:** Comparison of HHV detection methods.

Method	Target viruses	Detection limit	Detection time	Equipment requirement	References
PCR	Eight HHVs	10^4^ copies	Around 2 h	Thermal cycler	This study
RPA	Four HHVs	10^3^ copies	15–20 min	Simple heater	This study
RPA-LFA	HHV-6, HCMV	10^3^ copies	20 min	Simple heater	This study
PCR	Six HHVs	20 copies	Around 2 h	Thermal cycler	Tanaka et al. (2009)
PCR	Four HHVs	-	Around 2 h	Thermal cycler	H et al. (2021)
RPA-LFA	EBV	10^4^ copies	20 min	Simple heater	Sun et al. (2024)
dPCR	Eight HHVs	-	Around 2 h	Specialized platform	Lee et al. (2021)

Of course, the three detection systems established in this study still have room for further optimization. First, for the established octoplex PCR detection system, only qualitative detection and rough quantification of the eight viruses have been performed. That is, the detectable copy number of human herpesviruses in a single 50 μL reaction is 1 × 10^4^. Previous studies have reported that by designing a housekeeping gene as an internal reference and using two-dimensional probes, i.e., a 2D multiplex qPCR approach, it is possible to simultaneously detect all human herpesviruses in a single 25 μL reaction with a sensitivity of 30–300 copies (Li et al., 2021). Therefore, future work could consider incorporating probes and qPCR to achieve higher-resolution detection. Multiplex qPCR assays often require complex probe design and careful optimization to avoid cross-reactivity and signal interference, particularly when detecting multiple viral targets simultaneously. The primers developed in this study target conserved regions and are expected to be compatible with qPCR platforms. Future studies could further evaluate the performance of these primers in multiplex qPCR systems to expand their applicability in clinical and laboratory settings. In addition to qPCR, Lee et al. applied digital PCR technology to detect HHV DNA levels in saliva samples from clinical patients (Lee et al., 2021). While highly sensitive platforms for viral detection often require specialized instrumentation, higher costs, and more complex workflows, the systems developed in this study are designed to be accessible and suitable for use in resource-limited settings. A key area for future research will be to further improve and validate detection sensitivity under such conditions. Notably, a difference in analytical sensitivity was observed among targets, with HHV-7 showing a lower detection limit (1 × 10^3^ copies per reaction) compared to the other HHVs (1 × 10^4^ copies per reaction). This variation may reflect differences in primer binding efficiency and target sequence characteristics and is commonly observed in multiplex amplification systems. In addition, further optimization of primer design and reaction conditions may help to reduce variability and enhance assay uniformity and overall performance. Furthermore, different reagent conditions were required for the two quadruplex RPA systems, likely due to differences in primer composition, amplification efficiency, and interactions among primer sets in multiplex reactions. Given that RPA is highly sensitive to primer competition and reaction balance, future work may focus on simplifying and standardizing reaction components, as well as exploring the feasibility of integrating targets into a single octuplex RPA system. Moreover, the lack of specialized RPA primer design software further increases the difficulty of optimization. Tan et al. (2022) provided a detailed analysis of the challenges of primer interference in multiplex RPA and suggested using statistical experimental designs, such as orthogonal experiments, to optimize primer combinations and reduce non-specific amplification (Tan et al., 2022). In subsequent experiments, additives such as betaine or polyethylene glycol could also be considered to enhance interactions between DNA and the enzyme complex, thereby improving specificity and amplification efficiency (Li et al., 2018). Furthermore, it should be noted that all performance evaluations in this study were conducted under controlled laboratory conditions using plasmid standards or cultured viral isolates. When applied to complex clinical matrices, such as whole blood or cerebrospinal fluid, additional challenges may arise. These include the presence of amplification inhibitors, variability in nucleic acid extraction efficiency, and differences in viral load and sample quality, all of which may affect assay performance.

Finally, the currently established duplex RPA-LFA system can detect HCMV and HHV-6 simultaneously, but it still has the potential to detect more than two viruses using lateral flow strips. Although this study referred to Wang et al. (Wang et al., 2021) to eliminate false positive results by introducing nfo probes in avian influenza virus detection, the introduction of probes makes the multiplex detection system more complex and increases the difficulty of establishing the system. Therefore, adding detection for other human herpes viruses still requires a lot of screening and exploration. At present, while multiplex RPA-LFA has been widely explored in other pathogen detection areas such as bacterial or foodborne pathogen monitoring (Jin et al., 2023), in future experiments, the gold nanoparticles in the strips can be replaced with more interference-resistant europium nanoparticles to adapt to multiplex detection in complex samples. The strip reader can measure the signal intensity and convert it into T/C values for quantitative analysis. Meanwhile, further optimization of experimental conditions can aim to construct quadruplex or even higher multiplex RPA-LFA detection systems to achieve rapid visual detection of multiple human herpes viruses.

In conclusion, the octoplex PCR detection system, the two quadruplex RPA detection systems, and the duplex RPA-LFA detection system established in this study meet various detection needs from the laboratory to the field. They achieve efficient and sensitive detection of multiple human herpes viruses. These systems are especially suitable for screening human herpes virus contamination in cell banks and are important for quality control in cell culture. In addition, the experimental approach of this study provides a theoretical reference for the screening of other latent pathogens. It also has positive significance for promoting the biopharmaceutical industry toward safer and more efficient development.

## Data Availability

The original contributions presented in the study are included in the article/Supplementary Material, further inquiries can be directed to the corresponding authors.
