# Supplementary material for: Development of rapid multiplex human herpesvirus detection systems based on recombinase polymerase amplification and a lateral flow assay
Source: Front Cell Dev Biol. 2026 May 26;14:1751135. doi: 10.3389/fcell.2026.1751135 (PMC13246701; doi:10.3389/fcell.2026.1751135)
Supplement: Supplementary file 1 [file DataSheet1.docx]

**Supplementary Material**

**Table 1 Cell lines used for experiments**

| **Number** | **Cell line** | **Cell type** | **HHV status** |
| --- | --- | --- | --- |
| 1 | 104C1 | Mouse fibroblast |  |
| 2 | 143B/TK- | Human osteosarcoma |  |
| 3 | 293 | Human embryonic kidney |  |
| 4 | 293T | Human embryonic kidney |  |
| 5 | 3T3-L1 | Mouse preadipocyte |  |
| 6 | 4T1 | Mouse mammary tumor |  |
| 7 | 769-P | Human renal carcinoma |  |
| 8 | 786-O | Human renal carcinoma |  |
| 9 | A-204 | Human rhabdomyosarcoma |  |
| 10 | A-431 | Human epidermoid carcinoma |  |
| 11 | A549 | Human lung epithelial |  |
| 12 | A875 | Human melanoma |  |
| 13 | A9 | Mouse fibroblast |  |
| 14 | ACHN | Human renal carcinoma |  |
| 15 | Aedes albopictus clone C6/36 | Mosquito  (Aedes albopictus) |  |
| 16 | AN3 CA | Human endometrial carcinoma |  |
| 17 | Anglne | Human ovarian cancer |  |
| 18 | ARH-77 | Human B lymphocyte | EBV positive |
| 19 | B16-F0 | Mouse melanoma |  |
| 20 | B16-F1 | Mouse melanoma |  |
| 21 | B6YH4 | Mouse hybridoma |  |
| 22 | B82 | Mouse fibroblast |  |
| 23 | B95-8 | Marmoset B lymphocyte | EBV positive |
| 24 | BALB/3T3 clone A31 | Mouse fibroblast |  |
| 25 | BCBL-1 | Human B lymphoma | HHV-8 positive |
| 26 | BEAS-2B | Human bronchial epithelial |  |
| 27 | BeWo | Human placental trophoblast |  |
| 28 | BHK21 | Hamster kidney fibroblast |  |
| 29 | BS-C-1 | Monkey kidney epithelial |  |
| 30 | BT | Newborn bovine nasal epithelial |  |
| 31 | BV-173 | Human B leukemia |  |
| 32 | C-33 A | Human cervical carcinoma |  |
| 33 | CA46 | Human Burkitt lymphoma |  |
| 34 | HeLa | Human cervical epithelial |  |
| 35 | HUV-EC-C | Human endothelial | HHV-6 positive |

Table 2-1 Selection of PCR Primer Sequences

| **Virus** | **Primer** | | **Sequence (5′-3′)** | **Target fragment** |
| --- | --- | --- | --- | --- |
| HSV-1  NC_001806.2 | 1-FP1  1-RP1 | CCTCAACATACCCCGCTGTT  GCCATCGCACCAATACACAA | | 113 bp |
|  | 1-FP2  1-RP2 | CAAACCCAACCGTCCCGTAG  GCCATCGCACCAATACACAA | | 270 bp |
|  | 1-FP3  1-RP3 | CATGAAGCCCCCAACATGAC  GTCCCTTTGAGGTGAGTCGG | | 367 bp |
|  | 1-FP4  1-RP4 | CAACCCCAACTCCAGACCAC  CCTTTGAGGTGAGTCGGGTC | | 397 bp |
|  | 1-FP5  1-RP5 | TGGTTCTTGTCGGTGTATCGG  GTCGTCCCTTTGAGGTGAGT | | 453 bp |
| HSV-2  NC_001798.2 | 2-FP1  2-RP1 | CCCGGTACGCTCTCGTAAAT  CCCACCTCTACCCACAACAG | | 124 bp |
|  | 2-FP2  2-RP2 | GTGACGTACTACCGGCTCAC  ATTTACGAGAGCGTACCGGG | | 159 bp |
|  | 2-FP3  2-RP3 | CTTCGGCAGTATGGAGGGTG  CTACCCACAACAGACCCACG | | 211 bp |
|  | 2-FP4  2-RP4 | TATGCCTATCCCCGGTTGGA  ACACCCTCCATACTGCCGAA | | 259 bp |
|  | 2-FP5  2-RP5 | ACGTCCTCCGATTCGCCTA  GTTTTCGCTGCGGAGGC | | 290 bp |
|  | 2-FP6  2-RP6 | TATGCCTATCCCCGGTTGGA  CTACCCACAACAGACCCACG | | 439 bp |

Table 2-2 Selection of PCR Primer Sequences

| **Virus** | **Primer** | **Sequence (5′-3′)** | **Target fragment** |
| --- | --- | --- | --- |
| VZV  NC_001348.1 | 3-FP1  3-RP1 | GTCTAACATTAACGCGCTACAACAA  ATGGTTGTTCTCGGTCTGGAATAAT | 122 bp |
|  | 3-FP2  3-RP2 | CCCGCGATTTATGAAGATAGGTTAG  TAATCGGTGTCAGAATCTTCATCCC | 193 bp |
|  | 3-FP3  3-RP3 | CTAACATTAACGCGCTACAACAACC  TGTGTTTAAGTAGTCGTATTGGCTG | 254 bp |
|  | 3-FP4  3-RP4 | ACAGCCAATACGACTACTTAAACAC  TAATCGGTGTCAGAATCTTCATCCC | 326 bp |
|  | 3-FP5  3-RP5 | GTCTAACATTAACGCGCTACAACAA  CTAACCTATCTTCATAAATCGCGGG | 395 bp |
| EBV  NC_001847.1 | 4-FP1  4-RP1 | CTGCTTCCTTGTGCTCCTGCCGG  CTCCAAGAGCTCCCCGAAGCAACAG | 83 bp |
|  | 4-FP2  4-RP2 | GACAGGCTTAACCAGACTCATGTCAA  CACAAGGAAGCAGGCGAGGCAAGAA | 85 bp |
|  | 4-FP3  4-RP3 | TTCTTGCCTCGCCTGCTTCCTTGTG  CTCCAAGAGCTCCCCGAAGCAACAG | 95 bp |
| HCMV  NC_006273.2 | 5-FP1  5-RP1 | GAGGTCTTCAGAACAAAACGGAAGA  CACGGTCAGGTTGTAACAAGAGTAATA | 368 bp |
|  | 5-FP2  5-RP2 | ACGTGTTACTGGCGGAGTCG  TTGAGTGTGGCCAGACTGAG | 257 bp |
| HHV-6  NC_001664.4 | 6-FP1  6-RP1 | GATATGCACTCACCGGGAGC  TGAGCGTACCACTTTGCAGG | 125 bp |
|  | 6-FP2  6-RP2 | TTAAGAGCTTACGGACAGGTT  GTCCTATCTGTACATGGTCGTT | 142 bp |
|  | 6-FP3  6-RP3 | GATATGCACTCACCGGGAGC  CCCTGATTTCCGTTGTGTGTT | 223 bp |
|  | 6-FP4  6-RP4 | TAGATGATTGGTGCACGTATGC  CTAATGTCTCTTCGTATCCACG | 334 bp |
|  | 6-FP5  6-RP5 | ATCCGAGTGATGATTTCTGGACTAA  GGCGTTCCCGTCGAAGAAA | 398 bp |

Table 2-3 Selection of PCR Primer Sequences

| **Virus** | **Primer** | **Sequence (5′-3′)** | **Target fragment** |
| --- | --- | --- | --- |
| HHV-7  NC_001716.2 | 7-FP1  7-RP1 | CTCTGCGATCTATCACTAGTCACTT  TAATGCTAATGCTCTCTACGTTCCA | 114 bp |
|  | 7-FP2  7-RP2 | AATAGAAAACACTTTGCACGTTGGA  AGTAAACGAAATAACCGTCACGATG | 196 bp |
|  | 7-FP3  7-RP3 | CATCGTGACGGTTATTTCGTTTACT  TCCATACAAAGAAGAATTCCAGGGT | 385 bp |
| HHV-8  NC_009333.1 | 8-FP1  8-RP1 | GCACTCGACAAGAGTATAGTGGTTA  TATTTTGTAAACGGTGGCAATCTCC | 121 bp |
|  | 8-FP2  8-RP2 | TACCTTTGTATCCTATCAGCCTTGG  ACTCTGAAGATAGGAGAACATGACG | 198 bp |
|  | 8-FP3  8-RP3 | GATCCCTCTGACAACCTTCAGATAA  AAGTTCCGCCATATTTACATCATCC | 261 bp |
|  | 8-FP4  8-RP4 | GGAGATTGCCACCGTTTACAAAATA  GTGGTATATAGATCAAGTTCCGCCA | 437 bp |

Table 3-1 Pentaplex PCR reaction system

| **Reagent** | **Volume (μL)** |
| --- | --- |
| 2 ×TaKaRa Ex Premier^TM^ DNA Polymerase Dye plus | 25 |
| FP（10 μM）  RP（10 μM）  Template  ddH_2_O | 2.5  2.5  5  15 |

Table 3-2 Hexaplex PCR reaction system

| **Reagent** | **Volume (μL)** |
| --- | --- |
| 2 ×TaKaRa Ex Premier^TM^ DNA Polymerase Dye plus | 25 |
| FP（10 μM）  RP（10 μM）  Template  ddH_2_O | 3  3  6  13 |

Table 3-3 Heptaplex PCR reaction system

| **Reagent** | **Volume (μL)** |
| --- | --- |
| 2 ×TaKaRa Ex Premier^TM^ DNA Polymerase Dye plus | 25 |
| FP（10 μM）  RP（10 μM）  Template  ddH_2_O | 3.5  3.5  7  11 |

Table 3-4 Octaplex PCR reaction system

| **Reagent** | **Volume (μL)** |
| --- | --- |
| 2 ×TaKaRa Ex Premier^TM^ DNA Polymerase Dye plus | 25 |
| FP（10 μM）  RP（10 μM）  Template  ddH_2_O | 4  4  8  9 |

Table 4-1 GenDx ERA Kit

Quadruple RPA Reaction System

| **Reagent** | **Volume (μL)** |
| --- | --- |
| Freeze-dried reaction pellets | --- |
| Dissolving solution | 20 |
| FP（20 μL） | 2 |
| RP（20 μL） | 2 |
| Template | 4 |
| ddH_2_O | 20 |
| Activator | 2 |

Table 4-2 TwistAmp® Basic kit

Quadruple RPA Reaction System

| **Reagent** | **Volume (μL)** |
| --- | --- |
| Freeze-dried reaction pellets | --- |
| Rehydration buffer | 29.5 |
| FP（20 μL） | 2 |
| RP（20 μL） | 2 |
| Template | 4 |
| ddH_2_O | 10 |
| Activator | 2.5 |


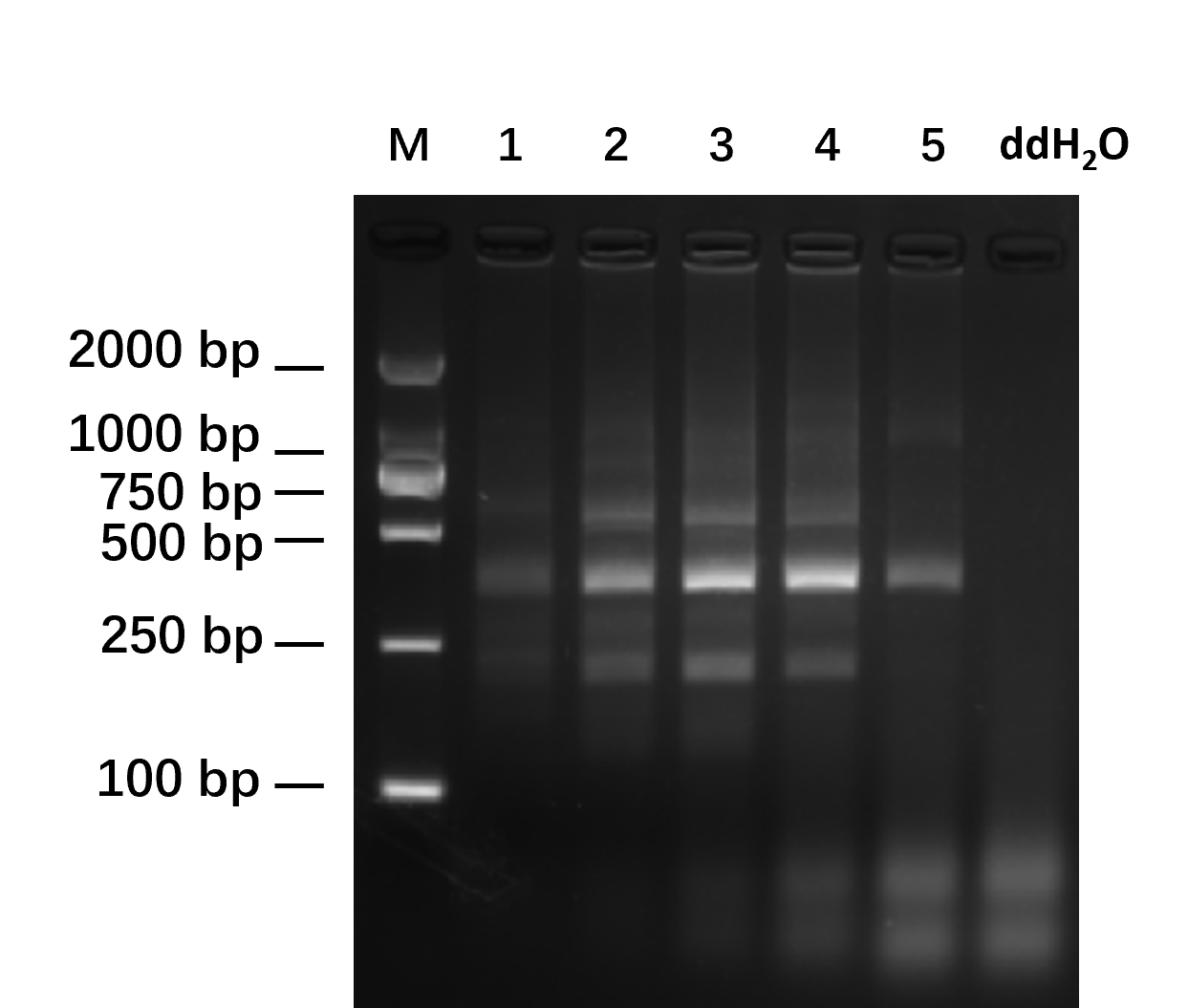


**Figure S1. Attempts to construct the octuple RPA detection system.**

1–5: total primer concentrations of 100, 200, 300, 400, and 500 nM, respectively; M: 2000 bp DNA marker.
